# Bidirectionality Between Perceived Immediate and Long-Term Benefits and Losses and Internet Gaming Disorder Among Chinese Adolescent Gamers: Prospective Longitudinal Study

**DOI:** 10.2196/74030

**Published:** 2026-02-26

**Authors:** Siman Li, Jianxin Zhang, Ji-bin Li, Joseph TF Lau, Yanqiu Yu

**Affiliations:** 1 Department of Preventive Medicine and Health Education, School of Public Health, Fudan University, Dong'an Road 130, Xuhui District, Shanghai, 200032, China, 862154237707; 2 West China School of Public Health, Sichuan University, Chengdu, Sichuan, China; 3 Department of Clinical Research, State Key Laboratory of Oncology in South China, Collaborative Innovation Center for Cancer Medicine, Sun Yat-sen University Cancer Center, Guangdong, Guangdong, China; 4 Public Mental Health Center, School of Mental Health, Wenzhou Medical University, Wenzhou, China; 5 Zhejiang Provincial Clinical Research Center for Mental Disorders, Affiliated Wenzhou Kangning Hospital of Wenzhou Medical University, Wenzhou, China

**Keywords:** internet gaming disorder, time perspective, perceived benefits, perceived losses, cross-lagged panel model, adolescents

## Abstract

**Background:**

Adolescents perceive both immediate and long-term benefits and losses related to internet gaming, affecting their risk of internet gaming disorder (IGD). These perceptions could also be shaped and reinforced by IGD, indicating potential bidirectionality.

**Objective:**

This study aimed to investigate the bidirectional relationships between perceived immediate and long-term benefits in 3 domains (mental health, social relationships, and personal achievement) and IGD, and between perceived immediate and long-term losses in 6 domains (mental health, sleep quality, academic performance, family relationships, social relationships, and personal achievement) and IGD.

**Methods:**

A 12-month 2-wave prospective longitudinal study was conducted among junior middle school students who had played internet games in the past 12 months in Guangzhou and Chengdu, China, with a baseline survey (T1, December 2018) and the other identical follow-up survey conducted 1 year later (T2, December 2019). The participating schools were conveniently selected; all Grade 7 and 8 students were invited to self-administer the questionnaires in a classroom setting without the presence of the schoolteachers. The final sample size was 1173 students (mean age 12.5, SD 0.6 y; male: 693/1173, 59.1%). IGD was assessed by using the 9-item *Diagnostic and Statistical Manual of Mental Disorders, Fifth Edition* IGD checklist.

**Results:**

Cross-lagged panel analysis (adjusting for background factors) showed (1) stronger perceived immediate benefits of mental health (*β*=.08, 95% CI 0.01-0.15) and personal achievement (*β*=.10, 95% CI 0.01-0.20) at T1 significantly predicted more IGD symptoms at T2; (2) more IGD symptoms at T1 significantly predicted stronger perceived immediate and long-term benefits of social relationships (immediate: *β*=.09, 95% CI 0.03-0.15; long-term: *β*=.11, 95% CI:0.05-0.17) and personal achievement (immediate: *β*=.12, 95% CI 0.06-0.18; long-term: *β*=.10, 95% CI 0.04-0.16) at T2; (3) more IGD symptoms at T1 significantly predicted stronger perceived immediate and future losses in mental health (immediate: *β*=.09, 95% CI 0.03-0.15; long-term: *β*=.08, 95% CI 0.02-0.14), sleep quality (immediate: *β*=.10, 95% CI 0.04-0.16; long-term: *β*=.13, 95% CI 0.07-0.19), academic performance (immediate: *β*=.09, 95% CI 0.04-0.15; long-term: *β*=.07, 95% CI 0.01-0.13), and family relationships (immediate: *β*=.11, 95% CI 0.05-0.17; long-term: *β*=.10, 95% CI 0.04-0.16) at T2, as well as perceived long-term losses in social relationships at T2 (*β*=.08, 95% CI 0.02-0.14).

**Conclusions:**

This study was innovative in integrating time perspective into both perceived benefits and losses of internet gaming, a cognitive dimension previously overlooked in literature. The current findings advance the field by revealing the unidimensional predictive effects of IGD on perceived immediate and long-term benefits and losses, with 2 exceptions of perceived immediate and long-term benefits of mental health and personal achievement conversely predicting IGD. These results contribute to the development of effective interventions: the cognitive components should go beyond the general pros and cons of gaming and target the potential temporal bias gamers hold.

## Introduction

Internet gaming is increasingly popular; the number of internet gamers worldwide reached 3.31 billion in 2023, with a growing rate of 4.3% year-on-year [1]. China had the largest number of internet gamers (668 million as of December 2023) [2]. The internet penetration rate of Chinese minors (aged below 18 y) was 97.2%, and 67.8% of them played internet games regularly [3]. Internet gaming could lead to internet gaming disorder (IGD), a mental disorder included by *the International Classification of Diseases, 11th Revision (ICD-11*) [4], and negative consequences of social (eg, poor parent-child relationships), psychological (eg, depression), and functional (eg, impaired cognitive functions) problems [5-14]. A meta-analysis of 96 studies reported the pooled prevalence of IGD among adolescents ranged from 7.5% to 10% globally [15]; it ranged from 2.4% to 21.5% in China [16,17]. The potentially high adolescent IGD prevalence underscores the need for a better understanding of the precedents and consequences of IGD.

Numerous theoretical and empirical studies highlight the role of gaming-specific cognitions in understanding IGD development and maintenance [18], among which outcome expectancy forms a typical domain. According to social cognitive theory, outcome expectancy refers to tangible or intangible outcomes anticipated as results of a behavior [19]. Outcome expectancy could be manifested in positive (ie, perceived benefits) and negative (eg, perceived losses) domains [19]. Regarding perceived benefits of internet gaming, a recent study summarized 3 key dimensions: mental health (eg, emotional boost), social relationships (eg, social interactions), and personal achievement (eg, improved self-esteem) [20]; all these perceived benefits were positively associated with IGD in multiple cross-sectional studies [21-26]. To our knowledge, these associations have not yet been demonstrated longitudinally. Regarding perceived losses due to internet gaming, internet gaming can entail potential negative consequences [27-30] that constitute losses to gamers. The literature generally identified 6 dimensions of these losses. First, IGD could exacerbate mental problems [5,8]. Second, IGD predicted strongly sleep problems (eg, sleep deprivation [6] and poor sleep quality [31,32]). Third, IGD had negative impacts on family relationships, including worsened parent-child relationships [33] and decreased family functioning [34]. Fourth, although internet gaming provides a platform for online social interactions [35], it could undermine real-life social interactions [36,37] and increase social anxiety or phobia [5,38-40], generating losses in social relationships. Last, internet gaming may lead to declines in academic performance and personal achievement. IGD was longitudinally associated with worsened academic achievement [41,42] and personal achievement [43,44]. Adolescent gamers may be aware of these losses, potentially changing their gaming behaviors and affecting IGD risk. Notably, while existing literature has examined associations between IGD and negative consequences, few studies have directly investigated perceived losses per se, their predictive effects on IGD, or the reverse association.

It is also novel to integrate the time perspective into the concept of perceived benefits and losses of internet gaming. The temporal frames of past, present, and future affect one’s decision-making and a variety of addictive behaviors [45-48]. Accordingly, perceived benefits and losses of internet gaming could occur at present (immediate benefits and losses) or in the future (long-term benefits and losses). Our literature review identified only 1 cross-sectional study looking at both perceived immediate and future benefits of internet gaming simultaneously, and it reported positive associations between these 2 perceived benefits and IGD with a comparable effect size [20]. It suggests that perceived future benefits might be as important as perceived immediate benefits and should not be neglected in future studies. In contrast, it was expected that these perceived benefits might be the outcomes of IGD. Our literature search identified a sole study reporting that IGD significantly predicted positive outcome expectancies of using internet gaming as a self-regulatory strategy [49]. In addition, no studies looked at the reciprocity between perceived immediate and long-term losses and IGD.

The reciprocal determinism construct of Social Cognitive Theory postulates that personal cognitive factors are key drivers of health behavior and could be shaped and reinforced by the behavior itself [19], supporting the bidirectional relationships between perceived immediate and long-term benefits and losses and IGD. There is additional theoretical support. The expectancy-value theories postulate that a positive outcome expectancy would increase the likelihood of performing a behavior, while a negative outcome expectancy would decrease the likelihood of performing the behavior [50]. Accordingly, perceived benefits would be expected to increase IGD risk while perceived losses were expected to mitigate it. Conversely, the predictive effect of IGD on perceived benefits and losses could be explained by Cognitive Dissonance Theory [51]. There may be cognitive dissonance when one’s behavior contradicts one’s well-being or values. To reduce this dissonance without changing behavior, the individual may alter their perceptions, potentially increasing perceived benefits and reducing perceived losses. Conversely, experiencing the negative consequences of IGD could plausibly reduce perceived benefits and heighten perceived losses.

Given the background, this 2-wave prospective longitudinal study aimed to (1) investigate the levels of perceived immediate and long-term benefits of internet gaming in 3 dimensions (mental health, social relationships, and personal achievement) and perceived immediate and long-term losses in 6 dimensions (mental health, sleep quality, academic performance, family relationships, social relationships, and personal achievement) among Chinese adolescents, and (2) examine the bidirectional relationships between perceived immediate and long-term benefits and losses and IGD. It was hypothesized that (1) perceived immediate and long-term benefits at baseline would be positively associated with IGD at follow-up, (2) perceived immediate and long-term losses at baseline would be negatively associated with IGD at follow-up, (3) IGD at baseline would be significantly associated with perceived immediate and long-term benefits and losses at follow-up, and the directions would be either positive or negative.

## Methods

### Participants and Data Collection

A 12-month 2-wave prospective longitudinal study was conducted among junior middle school students in Guangzhou and Chengdu, China; baseline and follow-up surveys were performed in December 2018 and 2019, respectively. The inclusion criteria at baseline included full-time grade 7 and grade 8 students, aged 12‐14 years, who had played internet games in the past 12 months at baseline, and were willing to participate in this survey. The selection of Grade 7 and 8 students was driven by their behavioral risks and environmental shifts specific to early adolescence. Behaviorally, this period involves heightened sensation-seeking and emotional instability, making the immediate gratification of gaming highly attractive [52]. Environmentally, these students are navigating the stressful transition to secondary education and experiencing reduced parental supervision regarding digital device use compared to primary school. Those Grade 9 students were excluded as they would leave school after 1 year and could not be followed up.

With the assistance of local education sectors, 4 and 2 junior middle schools were conveniently selected, and all grade 7 and 8 students of the selected schools were invited for participation; the in-school surveys were approved by school principals. The anonymous survey was self-administered by the students in the classroom setting in the absence of schoolteachers. Before the survey, well-trained field workers prebriefed the students on the objectives, content, logistics, and voluntary nature of this study, and that submitting a completed questionnaire implied informed consent for participation. Written informed consent was not collected to maintain anonymity. Such information was also printed on the cover page of the structured questionnaire. In addition, field workers assisted the students (eg, answering inquiries) and did the quality check during and after the survey, respectively. After 12 months, the students completed the same questionnaire with the same data collection procedures. The reporting of this study follows the STROBE (Strengthening the Reporting of Observational Studies in Epidemiology) guidelines [53], and the checklist is included in the supplementary material.

At baseline, 2463 completed the questionnaire, and a total of 77.4% (1906/2463) questionnaires were matched for both baseline (T1) and follow-up (T2) surveys. The lost to follow-up (530/1906, 21.8%) group was more likely than those followed-up to be older, Chengdu participants, male, not living with both parents, having father’s and mother’s educational levels of junior middle school or below, and having higher scores of IGD and some domains of perceived immediate and long-term benefits and losses (Table S1 in Multimedia Appendix 1). Among the matched questionnaires, 25.9% (494/1906) were excluded due to not having played internet games in the past 12 months at T1, 5.4% (103/1906) due to missing data in key variables (eg, gaming behaviors and IGD), and 7.1% (136/1906) due to age below12 or above 14 years. The final sample size was 1173 in this study.

### Measurements

#### Background Variables

Background information was collected, including age, sex, city of study, whether living with both parents, father’s and mother’s educational levels (junior middle school or below, senior middle school or equal, or college or above), and perceived family financial status (very good, good, moderate, poor, or very poor).

#### IGD

The 9-item *DSM-5* Checklist was used to screen for the presence of IGD symptoms in the past 12 months, including (1) preoccupation, (2) withdrawal symptoms, (3) tolerance, (4) inability to control internet gaming, (5) prioritizing internet games over other activities, (6) continuing to play internet games excessively despite psychosocial problems, (7) deceiving others about gaming time, (8) using internet games for escapism, and (9) impaired functions (eg, social and academic performance) due to internet gaming. All the items were rated with binary response options (0=No and 1=Yes). A total score was calculated by summing the responses, resulting in a range from 0 to 9; higher scores indicate a higher severity of IGD symptoms. The Chinese version of the *DSM-5* Checklist has been validated among adolescents in China with satisfactory reliability and validity indices [54]. In this study, the Cronbach *α* of this checklist at T1 and T2 was 0.75 and 0.77, respectively.

#### Perceived Immediate and Long-Term Benefits of Internet Gaming

Perceived immediate and perceived long-term benefits of internet gaming are assessed using the Perceived Overall Immediate Benefits Scale (POIBS) and the Perceived Overall Future Benefits Scale (POFBS; Fudan University), respectively, both of which were developed in Chinese adolescents and comprised 3 dimensions: mental health, social relationships, and personal achievement [20]. Perceived immediate benefits were rated as the extent to which the participants believed that internet gaming had positive impacts AT PRESENT on each of the above 3 dimensions (eg, “How much positive impact do you think internet gaming has AT PRESENT on your mental health?”). Perceived long-term benefits were rated as the extent to which the participants believed that internet gaming had positive impacts IN THE FUTURE on each of the above 3 dimensions (eg, “How much positive impact do you think internet gaming has IN THE FUTURE on your mental health?”). The 6 items were rated by using 5-point Likert scales (0=None to 4=extremely strong); higher scores indicate higher levels of perceived immediate and long-term benefits. The Cronbach *α* of POIBS and POFBS were 0.76/0.75 and 0.78/0.78 at T1/T2, respectively.

#### Perceived Immediate and Long-Term Losses Due to Internet Gaming

Overall, 6 dimensions of perceived losses due to internet gaming were considered in this study based on a thorough literature review and expert panel discussion, including (1) mental health, (2) sleep quality, (3) academic performance, (4) family relationships, (5) social relationships, and (6) personal achievement. Following previous publications and taking reference from the above POIBS and POFBS scale [20], perceived immediate losses were rated as the extent to which the participants believed that internet gaming had negative impacts AT PRESENT on each of the above 6 dimensions (eg, “How much negative impacts do you think internet gaming has AT PRESENT on your mental health?”), while perceived long-term losses were rated as the extent to which the participants believed that internet gaming had negative impacts IN THE FUTURE on each of the above 6 dimensions (eg, “How much negative impacts do you think internet gaming has IN THE FUTURE on your mental health?”). The 12 items were rated by using 5-point Likert scales (0=None to 4=extremely strong); higher scores indicate higher levels of perceived immediate and long-term losses. The Cronbach *α* of perceived immediate and long-term losses scales were 0.87/0.88 and 0.89/0.90 at T1/T2, respectively. Confirmatory factor analyses confirmed the 6-item structure of perceived immediate and long-term losses at both waves, demonstrating satisfactory model fit indices [perceived immediate losses (T1/T2): all factor loadings over 0.67/0.66 (all *P*<.001), comparative fit index (CFI)=0.97/0.97, Tucker-Lewis Index (TLI)=0.95/0.95, and standardized root mean squared residual (SRMR)=0.03/0.03; perceived long-term losses (T1/T2): all factor loadings over 0.71/0.69 (all *P*<.001), CFI=0.98/0.97, TLI=0.96/0.95, and SRMR=0.02/0.03].

### Data Analysis

Descriptive statistics and normality tests (via Kolmogorov-Smirnov and Shapiro-Wilk) were performed. Little’s Missing Completely at Random (MCAR) test was conducted to evaluate the pattern of missing data; Multiple Imputation was used to handle missing data. Paired-sample *t* tests were used to test for within-individual differences in levels of perceived immediate and long-term benefits and losses at both T1 and T2. Such data analysis was performed by using SPSS software (version 26.0; IBM Corp). A 2-sided *P* value <.05 was defined as statistically different.

Cross-lagged panel analyses with the maximum likelihood robust estimator were performed using Mplus 8.3; the analyses were conducted across 20 imputed datasets, and parameters were pooled according to Rubin’s rules. In total, 9 models were fit to examine the potential bidirectional associations between each pair of perceived immediate versus long-term benefits and losses and IGD. These models had 3 types of correlations: (1) cross-sectional correlations between variables at the same time point, (2) autocorrelations between the level of the same variable at both T1 and T2, and (3) cross-lagged correlations between the variable at T1 and the other variables at T2. To better reveal the cross-lagged correlations (eg, perceived short-term benefits of mental health on IGD), both cross-sectional correlations and autocorrelations, as well as background factors (ie, age, sex, city of study, whether living with both parents, parental educational levels, and perceived family financial status) at T1 were controlled. Satisfactory model fit indices of the models included CFI≥0.90, TLI≥0.90, and SRMR≤0.08 [55].

### Ethical Considerations

This study was approved by the Survey and Behavioral Research Ethics Committee of the Chinese University of Hong Kong (number SBRE-18‐430). Participation in this study was entirely voluntary. Prior to data collection, all participants were informed of the study objectives, procedures, and their rights as research participants. Given the anonymous nature of the survey, written informed consent was not obtained. Instead, informed consent was implied by the voluntary completion and submission of the questionnaire. Such information was also printed on the cover page of the structured questionnaire. All data were anonymous and no direct personal identifiers were collected. To enable longitudinal matching while preserving anonymity, instead, the last 4 digits of the father’s and mother’s phone numbers and the last phonetic letters of the father’s and mother’s given names were collected for matching purposes. No identifiable information about participants is hence possible in any part of this study or supplementary material. No financial or material incentives were provided to participants for their involvement in this study. In addition, parental approval was sought, and the parental opt-out procedure was exercised.

## Results

### Descriptive Statistics

Among all participants, the mean age was 12.5 (SD 0.6; range=12-14) years; 72.1% (846/1173) were from Guangzhou and 27.9% (327/1173) from Chengdu; over half (693/1173, 59.1%) were male; 15.6% (183/1173) did not live with both parents; 29.9% (350/1173) perceived that their family financial level was poor or very poor; nearly one-third of the participants’ fathers (361/1173, 30.8%) or mothers (335/1173, 28.6%) had received college or above education (Table 1). The mean IGD scores were 2.1 (SD 2.1; range=0-9) and 1.7 (SD 2.1; range=0-9) at T1 and T2, respectively.

**Table 1.** Frequencies and proportions of background variables in the 2-wave cross-lagged panel study on bidirectionality between perceived immediate and long-term benefits and losses and internet gaming disorder among adolescent internet gamers in Chengdu and Guangzhou, China (December 2018 to December 2019).

Frequencies and proportions	n (%) (N=1173)
City of study	
Guangzhou	846 (72.1)
Chengdu	327 (27.9)
Sex	
Male	693 (59.1)
Female	480 (40.9)
Living with both parents	
Yes	975 (83.1)
No	183 (15.6)
Missing data	15 (1.3)
Family financial status	
Very good or good	112 (9.5)
Moderate	709 (60.4)
Poor or very poor	350 (29.9)
Missing data	2 (0.2)
Father’s education level	
Junior middle school or below	427 (36.4)
Senior middle school or equivalent	316 (26.9)
College or above	361 (30.8)
Missing data	69 (5.9)
Mother’s education level	
Junior middle school or below	451 (38.4)
Senior middle school or equivalent	322 (27.5)
College or above	335 (28.6)
Missing data	65 (5.5)

#### Within-Individual Comparison of Perceived Immediate and Long-Term Benefits and Losses at Both T1 and T2

The results (Table 2) showed (1) among the 3 dimensions of perceived immediate versus long-term benefits, all within-individual differences (except that between perceived immediate and long-term benefits of personal achievement at T2) were statistically significant, but the effect sizes were small (Cohen *d* ranged from 0.06 to 0.12), and (2) among the 6 dimensions of perceived immediate versus long-term losses, all within-individual differences (except those regarding sleep quality and social relationships at T1) were statistically significant with small effect sizes (Cohen *d* ranged from 0.08 to 0.22).

**Table 2.** The within-individual comparisons in perceived immediate and long-term benefits and losses in the 2-wave cross-lagged panel study on bidirectionality between perceived immediate and long-term benefits and losses and internet gaming disorder among adolescent internet gamers in Chengdu and Guangzhou, China (December 2018 to December 2019).

Dimension	T1^a^, mean (SD)	Cohen *d*	*P* value	T2^b^, mean (SD)	Cohen *d*	*P* value
Perceived benefits						
Mental health	−0.08	.004	0.06	.04
Immediate impacts	1.4 (1)			1.5 (1)		
Long-term impacts	1.4 (1.1)	1.4 (1.1)
Social relationships	0.07	.01	0.09	.002
Immediate impacts	1.5 (1.3)			1.5 (1.2)		
Long-term impacts	1.5 (1.3)	1.4 (1.2)
Personal achievement	−0.12	<.001	−0.03	.30
Immediate impacts	1.1 (1.2)			1.1 (1.1)		
Long-term impacts	1.2 (1.3)	1.1 (1.2)
Perceived losses						
Mental health	−0.22	<.001	−0.2	<.001
Immediate impacts	1.1 (1.1)			1.3 (1.1)		
Long-term impacts	1.3 (1.2)	1.4 (1.2)
Sleep quality	−0.05	.13	−0.08	.01
Immediate impacts	1.1 (1.2)			1.2 (1.2)		
Long-term impacts	1.1 (1.3)	1.3 (1.3)
Academic performance	−0.08	.007	−0.08	.004
Immediate impacts	1.3 (1.2)			1.3 (1.2)		
Long-term impacts	1.3 (1.3)	1.4 (1.3)
Family relationships	0.03	.25	−0.1	.001
Immediate impacts	1 (1.2)			1 (1.3)		
Long-term impacts	0.9 (1.2)	1.1 (1.2)
Social relationships	−0.15	<.001	−0.13	<.001
Immediate impacts	0.7 (1)			0.8 (1)		
Long-term impacts	0.8 (1.1)	0.9 (1.1)
Personal achievement	−0.13	<.001	−0.14	<.001
Immediate impacts	0.8 (1.1)			0.9 (1.1)		
Long-term impacts	0.9 (1.2)	1 (1.2)	

a T1: assessment at baseline.

b T2: assessment at follow-up.

#### Cross-Lagged Panel Analysis

##### Bidirectional Relationships Between Perceived Immediate Versus Long-Term Benefits and IGD

**Figure 1.**
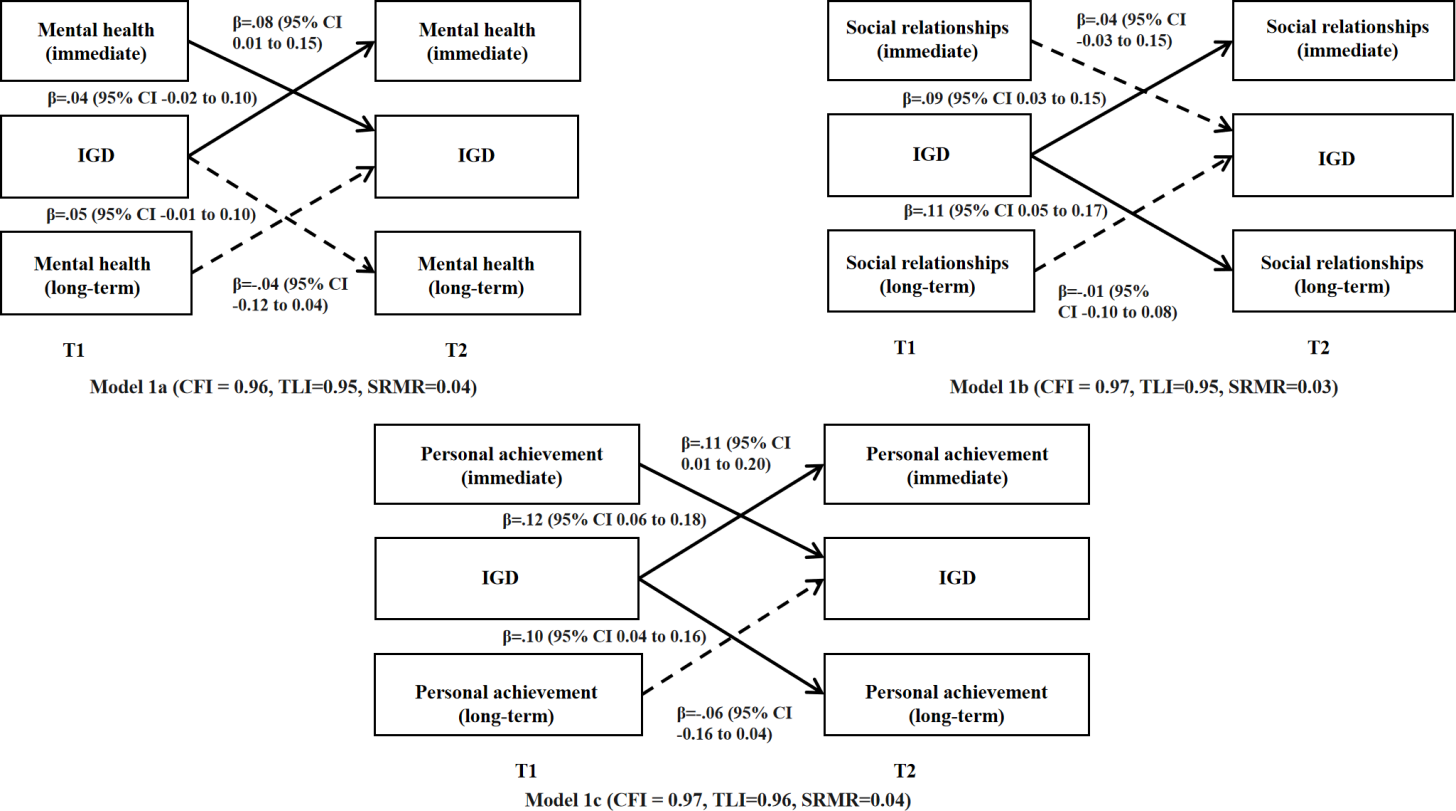
Cross-lagged panel model testing the bidirectional relationships between perceived immediate and long-term benefits and internet gaming disorder in the 2-wave cross-lagged panel study among adolescent internet gamers in Chengdu and Guangzhou, China (December 2018 to December 2019). Standardized coefficients were presented. Dashed lines represent nonsignificant paths and solid lines represent significant paths. The models were adjusted for background factors including the city of study, sex, age, father’s and mother’s educational level, and whether living with both parents. The cross-sectional correlations and autocorrelations were not presented for simplicity*.* CFI: Comparative Fit Index; IGD: internet gaming disorder; SRMR: standardized root mean square residual; T1: assessment at baseline; T2: assessment at follow-up; TLI: Tucker-Lewis Index.

Normality tests demonstrated that all key variables (perceived benefits and losses and IGD) at T1 and T2 violated the assumption of normality. Little’s MCAR test was statistically significant (*χ*^2^_1353_=1737.65; *P*<.001), suggesting that data were not missing completely at random. Consequently, the cross-lagged panel models were estimated using a maximum likelihood robust estimator combined with multiple imputation to account for nonnormality and missing patterns. In this section, 3 cross-lagged panel models were fit, each containing a pair of perceived immediate versus long-term benefits and IGD and adjusting for background variables (ie, age, sex, city of study, whether living with both parents, parental educational levels, and perceived family financial status); all models showed satisfactory model fit indices (Figure 1). Higher levels of perceived immediate benefits of mental health and personal achievement (but not social relationships) at T1 predicted more IGD symptoms at T2, while the predictive effects of perceived long-term benefits in the 3 dimensions on IGD were statistically nonsignificant. Conversely, more IGD symptoms at T1 significantly predicted higher levels of perceived immediate and long-term benefits of social relationships and personal achievement (but not mental health) at T2.

##### Bidirectional Relationships Between Perceived Immediate Versus Long-Term Losses and IGD

**Figure 2.**
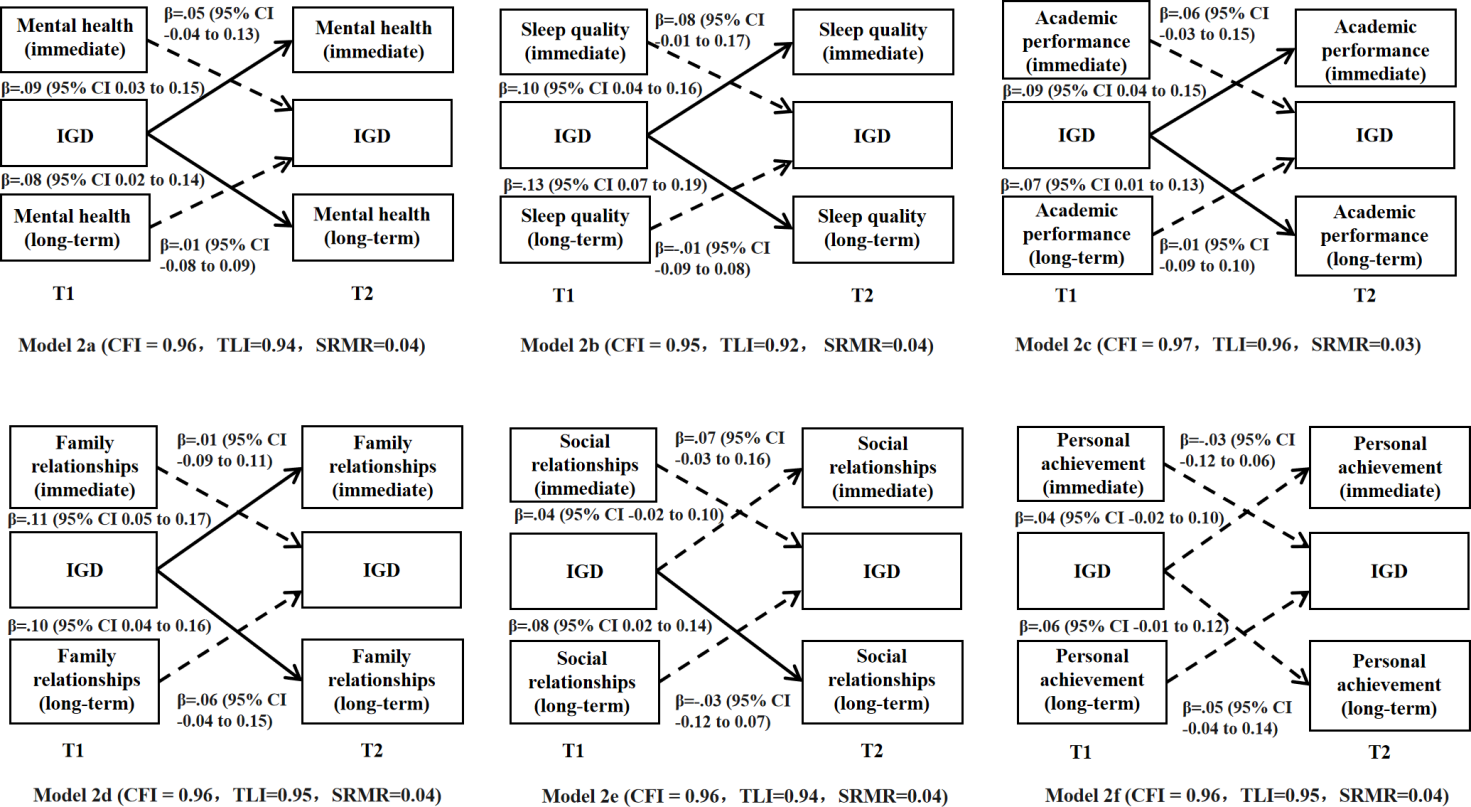
Cross-lagged panel model testing the bidirectional relationships between perceived immediate and long-term losses and internet gaming disorder in the 2-wave cross-lagged panel study among adolescent internet gamers in Chengdu and Guangzhou, China (December 2018 to December 2019). Standardized coefficients were presented. Dashed lines represent nonsignificant paths and solid lines represent significant paths. The models were adjusted for background factors including the city of study, sex, age, father’s and mother’s educational level, and whether living with both parents. The cross-sectional correlations and autocorrelations were not presented for simplicity. CFI: Comparative Fit Index; IGD: internet gaming disorder; SRMR: standardized root mean square residual; T1: assessment at baseline; T2: assessment at follow-up; TLI: Tucker-Lewis Index.

Similarly, 6 cross-lagged panel models were fitted, each containing a pair of perceived immediate versus long-term losses and IGD and adjusting for background variables; all models showed satisfactory model fit indices (Figure 2). All predictive effects of perceived immediate and long-term losses in mental health, sleep quality, academic performance, family relationships, social relationships, and personal achievement at T1 on IGD at T2 were statistically nonsignificant. Conversely, more IGD symptoms at T1 significantly predicted higher levels of perceived immediate and long-term losses in mental health, sleep quality, academic performance, family relationships, and social relationships (only the long-term losses) at T2; the predictive effect of IGD at T1 on perceived losses in personal achievement was statistically nonsignificant.

## Discussion

### Principal Findings

This study was novel to investigate the levels of perceived immediate and long-term benefits in 3 dimensions (mental health, social relationships, and personal achievement) and perceived immediate and long-term losses in 6 dimensions (mental health, sleep quality, academic performance, family relationships, social relationships, and personal achievement). All these levels were comparably low (within a range of 0‐4); the mean scores for perceived benefits ranged from 1.1 to 1.5, while those for perceived losses ranged from 0.7 to 1.4. These findings reflect the nature of the general adolescent sample, where the majority of participants engage in gaming recreationally without reporting extreme levels of both positive and negative experiences. Nonetheless, the observations confirmed a previous study reporting comparable levels of perceived immediate versus long-term benefits [22] and further underscore the importance of integrating the time perspective into perceived benefits and losses. As the first study to investigate perceived losses, our findings suggest that adolescents may perceive both benefits and losses equally, and perceived losses should be a nonnegligible target in future interventions; future studies in this regard are greatly warranted for confirmation and elaboration.

This longitudinal study was also the first to reveal the interesting bidirectional relationships between perceived immediate and long-term benefits and IGD. Although existing cross-sectional studies reported that perceived benefits of improved mental health, social relationships, and personal achievement were positively associated with IGD [21-26], this longitudinal study only partially supports these findings and revealed the differential predictive effects of these perceptions on IGD. The significant results highlight the importance of perceived immediate mental health and personal achievement benefits. Plausibly, the immediate mental health benefits could offer quick and easily accessible relief from negative emotions for gamers. Furthermore, the instant gratification model postulates that behaviors (eg, internet gaming) providing immediate emotional relief are more likely to be repeated [56], potentially contributing to increased IGD risk. On the other hand, the self-determination theory postulates that satisfaction with the psychological need for competence is powerful in motivating behavior [57]. Accordingly, the sense of accomplishment gained from internet gaming could supply immediate feedback and rewards (ie, immediate personal achievement benefits), motivating repetitive gaming, thereby increasing IGD risk. In addition, the significant predictive effects involving immediate but not long-term benefits corroborate previous publications emphasizing immediate rewards over delayed gratification in the context of IGD [58]. Speculatively, long-term benefits on mental health and personal achievement from internet gaming might occur gradually and remain less tangible to adolescent gamers, leading to the nonsignificant results. The nonsignificant predictive effects regarding perceived social relationships benefits might be associated with the complexity of social interactions, which can be context-dependent and influenced by external factors outside gaming (eg, peer relationships and behavioral inhibition) [59,60]. It is speculated that social interactions in gaming might not necessarily translate into meaningful or fulfilling relationships, which might reduce their impacts on IGD development.

Conversely, IGD at T1 significantly predicted perceived immediate and long-term benefits of social relationships and personal achievement at T2, but not those of mental health. It suggests that those with more IGD symptoms might amplify perceived benefits related to social relationships and personal achievement. Internet gaming is often designed with structured opportunities for social interactions (eg, multiplayer engagement) and measurable achievements (eg, leveling up) [61]; these aspects might be more pronounced among those with more IGD symptoms, leading to stronger perceptions of these benefits over time. In addition, those with more IGD symptoms may prioritize social and achievement benefits from internet gaming over those from other sources over time [21,62]. In contrast, individuals with more IGD symptoms might experience negative mental health outcomes, such as anxiety and depression [5,8]. These negative psychological conditions may overshadow the mental health benefits and lead to the nonsignificant predictive effect of IGD on perceived mental health benefits.

Unexpectedly, all predictive effects of perceived immediate and long-term losses in 6 domains at T1 on IGD at T2 were statistically nonsignificant. While perceived benefits and losses might be perceived at comparable levels, the psychological impacts of immediate benefits might have stronger influences on developing IGD due to their ability to fulfill psychological needs like competence as aforementioned [20]. This could overshadow the importance of perceived losses in terms of affecting IGD. This speculation was also supported by the concept of reinforcement that positive reinforcement (eg, perceived benefits) tends to have more immediate and stronger effects on motivating behavior in comparison with negative reinforcement or punishment (eg, perceived losses) [63]. Nonetheless, such speculations should be tested in future studies.

Reversely, this study observed differential predictive effects of IGD at T1 on the 6 domains of perceived immediate and long-term losses at T2. First, IGD significantly predicted perceived immediate and long-term losses in mental health, sleep quality, academic performance, and family relationships, suggesting that IGD could lead to noticeable harm in these domains. These findings align with empirical evidence that IGD cases face common negative consequences, such as depression, disturbed sleep patterns, worsened academic performance, and strained family relationships [27,33,41]. Notably, these domains of perceived losses might be more directly observable or harder to rationalize over time and are recommended to be emphasized in IGD intervention programs. Second, IGD at T1 significantly predicted perceived long-term, but not immediate, losses in social relationships, indicating a potential lag effect. It is speculated that, initially, the social benefits of internet gaming might buffer against perceived immediate losses, but, over time, those with more IGD symptoms might experience weakened offline social ties and relationships [64], making the long-term losses more apparent and significant. Last, the predictive effects of IGD at T1 on perceived immediate and long-term losses in personal achievement were statistically nonsignificant, suggesting that individuals with more IGD symptoms might not associate their gaming habits with detriments in personal achievement. Plausibly, as aforementioned, internet gaming may fulfill one’s psychological needs of competence [65] and provide satisfactory substitutes for real-life achievements [66], decreasing perceived losses.

The current findings have practical implications, as gaming-specific cognitions are important modifiable constructs in IGD interventions (eg, cognitive-behavioral therapy) [67]. IGD prevention interventions may focus more on the immediate benefits on mental health and personal achievement. For instance, alternatives for immediate rewards of mental health and personal achievements (eg, outdoor physical activities) should be provided for adolescents to reduce their reliance on and sensitivity toward these gaming rewards. It might also be helpful to separate gaming achievement from real-world achievement and then educate adolescents on how to fulfill the latter [68]. In contrast, IGD treatment intervention may underscore the cognitions of potential losses in mental health, sleep quality, academic performance, and family relationships, as well as the long-term losses in social relationships, while perceived rewards of social relationships and personal achievement are recommended to be downplayed.

There are several limitations of this study. First, the attrition rate of this study was about 20%. Attrition analysis revealed that those lost to follow-up demonstrated stronger perceived benefit and losses in some domains, besides specific background differences (eg, age and sex). In addition, Little’s MCAR test showed that the missing data were not completely random. It suggests that the study sample may have stronger perceived benefits and losses than the baseline population. Nonetheless, multiple imputation was used in the cross-lagged panel analyses to address the missing data patterns, increasing the robustness of the current findings. Furthermore, the study population was selected from 2 cities in China through convenience sampling, which may lead to selection bias. Cautions are needed when extrapolating the results to other regions or countries. Second, as participants were asked to self-administer the questionnaire, there might be reporting bias, including recall bias and social desirability bias. Third, although this study provides valuable insights on the bidirectional relationships between perceived immediate and long-term benefits and losses and IGD, it comprised only 2 time-points within a 12-month follow-up period. Future longitudinal studies with more time lags and longer study periods are needed to confirm the findings. In addition, while several predictive effects were statistically significant, their associated effect size was modest. It suggests that, despite statistical significance, these associations might have limited practical impacts on individuals. However, it is important to note that small effect sizes could still bear practical implications at the population level. Modest increases or reductions in each predictor might yield substantial benefits for the population, though future intervention studies are needed for confirmation. Last, there might be other potential losses due to internet gaming that were not included in this study (eg, losses in physical health and the financial domain).

### Conclusions

This study was novel in integrating the time perspective into perceived benefits and losses related to internet gaming and investigating their bidirectional relationships with IGD. The results are interesting in that only perceived immediate benefits of mental health and personal achievement predicted IGD, while IGD predicted perceived immediate and long-term benefits on social relationships and personal achievement, as well as perceived immediate and long-term losses in all 6 domains (except personal achievement). These findings enhance the understanding of the relationships between gaming-specific cognitions and IGD and shed new insights for relevant studies, taking into consideration the time perspective. The differential bidirectional relationships are also important for the design of IGD prevention and treatment interventions. Specifically, relevant cognitive components should target the specific temporal bias adolescent gamers possess, rather than focusing merely on the general benefits and losses of internet gaming.

## Supplementary material

10.2196/74030 Multimedia Appendix 1 Attrition analysis in the 2-wave cross-lagged panel study on bidirectionality between perceived immediate and long-term benefits/losses and internet gaming disorder among adolescent internet gamers in Chengdu and Guangzhou, China (December 2018 to December 2019).

## Abbreviations

CFI: comparative fit index
DSM-5: Diagnostic and Statistical Manual of Mental Disorders, Fifth Edition
ICD-11: *International Classification of Diseases, 11th Revision*
IGD: internet gaming disorder
MCAR: missing completely at random
POFBS: Perceived Overall Future Benefits Scale
POIBS: Perceived Overall Immediate Benefits Scale
SRMR: standardized root mean squared residual
STROBE: Strengthening the Reporting of Observational Studies in Epidemiology
TLI: Tucker-Lewis Index
