# Supplementary material for: Bidirectionality Between Perceived Immediate and Long-Term Benefits and Losses and Internet Gaming Disorder Among Chinese Adolescent Gamers: Prospective Longitudinal Study
Source: J Med Internet Res. 2026 Feb 26;28:e74030. doi: 10.2196/74030 (PMC12945363; doi:10.2196/74030)
Supplement: Multimedia Appendix 1 [file jmir-v28-e74030-s001.docx]

**Table S1.**

|  | Followed up  (n=1906) | | Lost to follow up  (n=530) | | | *P^#^* |
| --- | --- | --- | --- | --- | --- | --- |
|  | n/Mean | %/SD | | n/Mean | %/SD |  |
| **Background factors** |  |  | |  |  |  |
| City of study |  |  | |  |  | <.001 |
| Guangzhou | 1,193 | 62.6 | | 166 | 31.3 |  |
| Chengdu | 713 | 37.4 | | 364 | 68.7 |  |
| Age (years)^¶^ | 12.6 | 0.7 | | 12.8 | 0.7 | <.001 |
| Sex |  |  | |  |  | <.001 |
| Female | 940 | 50.2 | | 214 | 40.8 |  |
| Male | 932 | 49.8 | | 310 | 59.2 |  |
| Living arrangement |  |  | |  |  | <.001 |
| Others | 301 | 15.8 | | 133 | 25.1 |  |
| With both parents | 1,548 | 81.2 | | 385 | 72.6 |  |
| Missing data | 57 | 3.0 | | 12 | 2.3 |  |
| Father’s educational level |  |  | |  |  | <.001 |
| Junior middle school or below | 749 | 39.3 | | 271 | 51.1 |  |
| Senior middle school | 503 | 26.4 | | 149 | 28.1 |  |
| College or above | 524 | 27.5 | | 69 | 13.0 |  |
| Missing data | 130 | 6.8 | | 41 | 7.7 |  |
| Mother’s educational level |  |  | |  |  | <.001 |
| Junior middle school or below | 767 | 40.2 | | 270 | 50.9 |  |
| Senior middle school | 520 | 27.3 | | 137 | 25.8 |  |
| College or above | 490 | 25.7 | | 76 | 14.3 |  |
| Missing data | 129 | 6.8 | | 47 | 8.9 |  |
| Family financial status |  |  | |  |  | .55 |
| Good/very good | 507 | 26.6 | | 132 | 24.9 |  |
| Moderate | 1,167 | 61.2 | | 331 | 62.5 |  |
| Poor/very poor | 194 | 10.2 | | 60 | 11.3 |  |
| Missing data | 38 | 2.0 | | 7 | 1.3 |  |
| **Dependent and independent variables** |  |  | |  |  |  |
| IGD score^¶^ | 2.1 | 2.2 | | 2.6 | 2.5 | <.001 |
| ***Perceived Benefits*** |  |  | |  |  |  |
| Mental health |  |  | |  |  |  |
| Immediate impacts | 1.3 | 1.0 | | 1.4 | 1.2 | .13 |
| Long-term impacts | 1.3 | 1.1 | | 1.4 | 1.2 | .19 |
| Social Relationships |  |  | |  |  |  |
| Immediate impacts | 1.4 | 1.3 | | 1.4 | 1.3 | .93 |
| Long-term impacts | 1.4 | 1.3 | | 1.4 | 1.3 | .45 |
| Personal achievement |  |  | |  |  |  |
| Immediate impacts | 1.1 | 1.2 | | 1.2 | 1.3 | .006 |
| Long-term impacts | 1.1 | 1.3 | | 1.3 | 1.3 | .01 |
| ***Perceived Losses*** |  |  | |  |  |  |
| Mental health |  |  | |  |  |  |
| Immediate impacts | 1.2 | 1.2 | | 1.3 | 1.2 | .07 |
| Long-term impacts | 1.4 | 1.3 | | 1.5 | 1.4 | .41 |
| Sleep quality |  |  | |  |  |  |
| Immediate impacts | 1.2 | 1.3 | | 1.3 | 1.4 | .02 |
| Long-term impacts | 1.3 | 1.4 | | 1.4 | 1.4 | .02 |
| Academic performance |  |  | |  |  |  |
| Immediate impacts | 1.4 | 1.3 | | 1.5 | 1.3 | .08 |
| Long-term impacts | 1.5 | 1.4 | | 1.5 | 1.4 | .62 |
| Family Relationships |  |  | |  |  |  |
| Immediate impacts | 1.1 | 1.2 | | 1.2 | 1.3 | .03 |
| Long-term impacts | 1.1 | 1.3 | | 1.2 | 1.4 | .06 |
| Social Relationships |  |  | |  |  |  |
| Immediate impacts | 0.9 | 1.1 | | 1.1 | 1.2 | <.001 |
| Long-term impacts | 1.0 | 1.2 | | 1.2 | 1.3 | .002 |
| Personal achievement |  |  | |  |  |  |
| Immediate impacts | 1.0 | 1.2 | | 1.1 | 1.2 | .01 |
| Long Term Impacts | 1.1 | 1.3 | | 1.3 | 1.4 | .004 |

Note. IGD = Internet gaming disorder.

^#^, Chi-square test and independent-sample t-test were used when appropriate.

^¶^, Mean value and SD were reported for continuous variables.
